# Policy practice for narrowing urban–rural healthcare gaps: determinants and implementation path of the urban doctors servicing rural areas policy in Beijing, China

**DOI:** 10.3389/fpubh.2025.1456142

**Published:** 2025-09-22

**Authors:** Yao Liu, Chen Lu, Jiale Sheng, Yurou Zou

**Affiliations:** ^1^Beijing Xicheng District Zhanlanlu Hospital (Beijing First Rehabilitation Hospital), Beijing, China; ^2^School of Public Policy and Management, University of Chinese Academy of Sciences, Beijing, China; ^3^Sino-Danish College, University of Chinese Academy of Sciences, Beijing, China

**Keywords:** counterpart support policy, urban doctors servicing in rural hospitals (UDSR) policy, policy implementation, fuzzy-set qualitative comparative analysis, implementation path, Van Meter-Van Horn policy implementation framework

## Abstract

**Background:**

The urban–rural disparities in healthcare resources and service quality remain a critical challenge for equitable public health development in China. To address this, Beijing has implemented the “Urban Doctors Serving Rural Areas (UDSR)” policy-a key counterpart support initiative. Its core goal is to bridge urban–rural healthcare gaps by mobilizing urban medical professionals to serve rural institutions.

**Methods:**

Grounded in Van Meter and Van Horn’s policy implementation framework, this study employs fuzzy-set Qualitative Comparative Analysis (fsQCA). It examines 39 annual observations from 13 paired urban–rural hospitals in Beijing over 2017–2019. To capture temporal changes, the study applies time-series QCA techniques. It analyzes conditions including support project quantity, subsidy source diversification, prescription rights, professional matching, communication channels, and policy cognition, with rural medical technology improvement as the outcome variable.

**Results:**

Three critical determinants of UDSR policy effectiveness are identified: the number of support projects, diversified subsidy sources, and prescription rights for urban doctors in rural institutions. Four implementation pathways are uncovered: (1) goal-and-cognition-driven, (2) professional-matching-driven, (3) external-funding-driven, and (4) comprehensive-factor-driven. Temporal analysis reveals two evolutionary paths: a dominant path achieved via integrating multiple factors, and a transitional path shifting from external funding reliance to professional alignment and goal cognition-reflecting adaptive maturation of the UDSR policy.

**Conclusion:**

This study advances understanding of counterpart support policy implementation by revealing synergistic combinations of influencing factors and dynamic evolutionary paths. The findings make two key contributions. Theoretically, they provide insights for policy implementation research in non-western contexts; practically, they offer guidance for optimizing urban–rural healthcare resource allocation and provide a framework for refining targeted support mechanisms in China and similar developing countries.

## 1 Proposing the problem

In rapidly developing economies—such as China, India, and Brazil—remarkable economic growth has often been accompanied by pronounced regional development disparities and socioeconomic inequalities. These disparities take multiple forms: for instance, within a single country, urban and rural areas may differ significantly in income distribution, healthcare access, education quality, and infrastructure conditions. Regional development disparities underscores the need for targeted policy interventions to reduce such socioeconomic inequalities (1). A promising strategy that is gaining attention is the concept of counterpart support or paired assistance policy (2)—a model where more developed regions provide resources, expertise, and services to less developed ones to narrow the regional disparities or address major public crises (3–7). China, which faces significant regional disparities, usually adopts counterpart support policies as key governance measures (8–10). These policies aim to address the imbalances through targeted assistance from more developed regions to less developed ones.

In the medical services field—critical to improving a nation’s public well-being—a notable gap in medical resources allocation persists between urban and rural areas across many countries (11). In China, rural areas often suffer from inadequate medical development, which reflects the broader imbalance and insufficiency in national health progress (12). There exist significant disparities in public services within metropolises (13). The significant decrements in urban–rural disparities and equitable distribution of health care utilization call for further reform by policymakers (14). To address the healthcare challenges in rural areas, China’s central government issued the “Decision of the Central Committee of the Communist Party of China and the State Council on Further Strengthening Rural Health Work.” This decision explicitly proposes increasing support for farmers and agriculture through health programs and poverty alleviation efforts, and mandate the participation of large/medium-sized urban medical institutions and the military in the “counterpart support” program (15). In alignment with the directives of the central government, local governments have initiated various measures according to local context. The government’s policy design aims to narrow the gap in public health-care services between urban and rural areas and to address the issues of inadequate development in the country (16). However, the reality often falls short of expectations: in China, the implementation of such policies is frequently more of a political mission than a substantive effort to drive development (17, 18). The effectiveness of urban–rural hospital counterpart support initiatives remains to be further improved (19, 20).

The potential impact of policies targeting health equity depends on both their design and implementation —two aspects that require ongoing evaluation and stakeholder engagement (21). Policy implementation involves multiple stakeholders, each with distinct social-psychological and interest-based needs—which make it hard to harmonize their relations (22, 23) Successful policy implementation needs collaborative behaviors of frontline agencies (1). Taking Beijing as an example, doctors and managers of both urban and rural hospitals encountered multiple problems during the policy implementation process. Most existing articles on urban–rural medical counterpart support focus on experience summaries and problem identification (24, 25), with insufficient in-depth scientific and theoretical analysis (26). Specifically, few studies examine the effects and influencing factors of this support from a policy implementation perspective. Academia’s understanding of counterpart support policies also lags behind their practical development (27).

Based on the aforementioned issues, this study aims to address the following questions: from the perspective of policy implementation, how can we identify the influencing factors that affect the effectiveness of counterpart support policies? How do these factors interact to form various pathways that impact policy effectiveness? As policy implementation and the counterpart support initiatives progress, have the key factors influencing policy effectiveness evolved over time?

To address these questions, this paper takes Beijing’s “urban doctors serving rural grassroots” policy as a case study. It constructs an analytical framework for urban–rural medical personnel support from the perspective of policy implementation analysis. Data were collected via on-site interviews and questionnaires with counterpart support hospitals, covering 13 pairs of counterpart support cases over three consecutive years. The study then use fuzzy set qualitative comparative analysis (fsQCA) to explore the key factors and implementation pathways affecting the policy’s effectiveness. The study aims to provide a theoretical basis for further refining and implementing policies for the allocation of medical resources. Furthermore, it seeks to serve as a valuable reference for urban–rural counterpart support endeavors in other regions.

## 2 Literature review and theoretical analysis

### 2.1 Literature review

The counterpart support framework represents a collaborative governance mechanism characterized by strong coordination from national authorities, generally led by the central government with the active involvement from local authorities (28). The supporting entities mainly provide assistance through personnel and technical deployment, mobile medical services, remote training and demonstrations programs, adoption of new technologies and initiatives, and the professional development of healthcare practitioners (29). The evolution of China’s counterpart support system is influenced by various factors, such as macro institutional structure, actors’ beliefs and strategies, critical junctures, and institutional ambiguity (30). Counterpart support was initially rooted in administrative mobilization, but has since evolved into a compound value rationality (31).

Medical counterpart support has played a crucial role in addressing the problem of regional imbalanced development and responding to sudden events, especially in achieving remarkable results in the prevention and control of the COVID-19 epidemic (10). However, in daily urban–rural medical counterpart support, evaluations of existing policies’ implementation effectiveness fall into two distinct perspectives. Some studies argue that urban–rural counterpart support has facilitated rural residents’ access to medical care (32), improved the service level of grassroots medical institutions (33), and strengthened county-level hospital construction (34). In Beijing, the medical counterpart support improved rural hospitals in terms of medical safety, and capacity to treat emergency cases and more diverse illnesses (35). However, other research underscores challenges such as heavy reliance on local government funding, leading to an increased local financial burden (36); obstacles in transferring doctors from higher-tier hospitals (37). Furthermore, the medical staff sent by urban hospitals to the counterpart support hospitals have not been effectively and reasonably utilized, which has caused personnel allocation tension in assisted hospitals (38).

Research of medical counterpart support mainly revolves around five aspects: the implementation carriers, talent management strategies, governing policies, current situation analysis, and case studies of effective counterpart support (26). Academia has not reached a consensus on the criteria for evaluating the efficacy of medical counterpart support. Some scholars propose an evaluation framework encompassing four dimensions: medical services provision, quality assurance and safety measures, sustainable development, and societal benefits (39). Conversely, others propose a comprehensive evaluation framework for urban–rural hospital counterpart support, including five indicators: health-care resource inputs, medical service levels, hospital management, operation management, and social benefits (40). Distinct characteristics and dominant factors emerge in different development stages, with research themes highly correlated with policy dynamics and grassroots needs (26). Studying medical counterpart support policies through the lens of policy tools unveils stage-specific characteristics. With a declining reliance on authoritative and incentive-based policy tools, there has been a concurrent increase in the use of capacity-building policy tools. Symbolic admonishment policy tools exhibit relative stability over time (41).

In summary, existing literature has analyzed medical counterpart support policies from multiple perspectives. However, research primarily relies on empirical generalization and lessons from past experiences. Relatively few studies have discussed the specific factors influencing policy outcomes in medical counterpart support. There is a lack of research on the implementation pathways for the combined effect of multiple influencing factors, and exploration of how these factors change over time remains limited.

### 2.2 Analytical framework

This study adopts the Van Horn-Van Meter model as the theoretical framework for analyzing the policy of sending urban doctors to serve in the rural hospitals (UDSR).

#### 2.2.1 Theoretical basis

Originally distilled from U.S. federal programmes, the model posits six interacting determinants—policy standards and objectives, resources, inter-organizational communication, enforcement activities, characteristics of implementing agencies, and the broader socio-economic environment—that jointly shape implementation outcomes (42). Owing to its parsimony and comprehensive coverage, the framework has become a textbook benchmark for diagnosing implementation gaps.

#### 2.2.2 Model selection rationale

This framework was selected mainly due to its high compatibility with the institutional context and research focus of this study: firstly, this policy possess a top-down policy attributes. The UDSR policy in Beijing is a typical top-down intervention, formulated by the municipal government in response to central mandates. Van Meter and Van Horn’s model, which emphasizes hierarchical administrative systems, directly addresses key elements in such policies (43). This aligns with our focus on how multi-level stakeholders interact in policy execution. Secondly, this policy features comprehensive factor coverage. The theoretical framework’s six dimensions precisely capture the complexity of UDSR policy implementation. Unlike narrower models, it allows us to examine both macro-level resources—for example, the subsidies diversification, and micro-level dynamics, such as physicians’ prescribing autonomy. These aspects are vital for discerning the synergistic factors that determine policy effectiveness. Thirdly, this model is contextually relevant to China’s governance. As noted in existing research, China’s counterpart support policies rely heavily on coordinated action across administrative levels (5, 9, 10). Van Meter and Van Horn’s emphasis on inter-organizational communication and resource dependencies provides a theoretical lens to unpack such coordination mechanisms in Beijing’s specific context.

#### 2.2.3 Six dimensions of the framework

##### 2.2.3.1 Policy standards and goals

Policy standards and goals are crucial determinants influencing the efficacy of policy implementation. Following the promulgation of the policy regarding urban doctors serving in rural areas in Beijing, clear assessment indicators were provided for supporting hospitals in each annual evaluation, aiming to promote the implementation of national rural revitalization work (44, 45). In the annual assessment of urban hospitals’ support for rural areas, the superior department evaluates the hospital’s paired support endeavors using 12 evaluation indicators. These criteria encompass various facets such as the quantity and duration of supporting personnel, the volume of diagnosis and treatment cases, participation in assisted learning activities, completion of surgical cases, demonstration surgeries conducted, consultations for challenging medical cases, academic rounds conduct, health checkups administered, delivery of academic lectures, provision of business training sessions, organization of free clinics, establishment of specialty departments, and the value of donated items.

##### 2.2.3.2 Policy resources

Policy implementation requires the allocation of pertinent resources. In the execution of the UDSR policy in Beijing, the allocation of human and financial resources has been tailored to meet stipulated requirements, ensuring the implementation of the policy and the achievement of its objectives (46). The human resources input in the medical field refers to the urban doctors who participate in rural medical service, while the financial resources are mainly used to subsidize various expenses associated with urban doctors’ service in rural areas.

##### 2.2.3.3 Implementation methods

Following the clarification of policy goals and evaluation criteria, the implementation of urban doctors to rural service policies involves communication and coordination among multiple organizational institutions and personnel. The first level of communication entails inter-institutional exchanges, such as communication between healthcare institutions and personnel management entities, as well as coordination between urban hospitals and rural hospitals. The second level of communication occurs within institutions, involving interactions between supporting health-care management personnel and various departments, as well as between departments and individual doctors. The processes and outcomes of these information exchanges and transmissions significantly affect the effectiveness of policy implementation.

##### 2.2.3.4 Characteristics of implementing institutions

The main entities of policy implementation institutions include urban and rural healthcare institutions. These institutions exhibit distinct characteristics, including hospital size, hospital level, medical service capacity, departmental resources, and the composition of healthcare technical personnel (47). Because of these varying attributes, different healthcare institutions adopt diverse assistance approaches in urban–rural healthcare counterpart support initiatives (48).

##### 2.2.3.5 System environment

The system environment of urban doctors serving in rural areas includes several dimensions: the political environment, economic environment, cultural environment, and socio-psychological environment (49).

The political environment primarily relates to the degree of attention from public media to the service of urban doctors in rural areas. This aspect signifies the level of societal awareness and discourse regarding the significance and impact of such initiatives.

The economic environment reflects the level of local economic development and the degree of economic incentives provided to urban doctors serving in rural areas. This includes subsidies, financial support mechanisms, and infrastructure investments aimed at facilitating urban–rural healthcare exchanges.

The cultural environment encompasses the customs, traditions, and societal norms prevalent in both urban and rural settings. It influences healthcare-seeking behaviors, Cognition of healthcare delivery, and the acceptance of urban doctors within rural communities.

The socio-psychological environment includes the habits, beliefs, and attitudes of various stakeholders, including administrators, doctors, patients, and community members. It encompasses factors such as trust in healthcare providers, Cognition of quality care, and the willingness to engage in collaborative healthcare initiatives. These environmental factors collectively shape the context in which urban doctors serve in rural areas, impacting the success and sustainability of such programs.

##### 2.2.3.6 Values of policy implementers

All aspects of the policy implementation process are dependent on different implementers. In this study, the principal implementers of the policy are managers and doctors in urban and rural hospitals. Significantly divergent perspectives and interest exist among these personnel (50). The Cognition and preferences of managers and doctors in urban hospitals have a substantial impact on policy implementation effectiveness.

**Figure 1:**
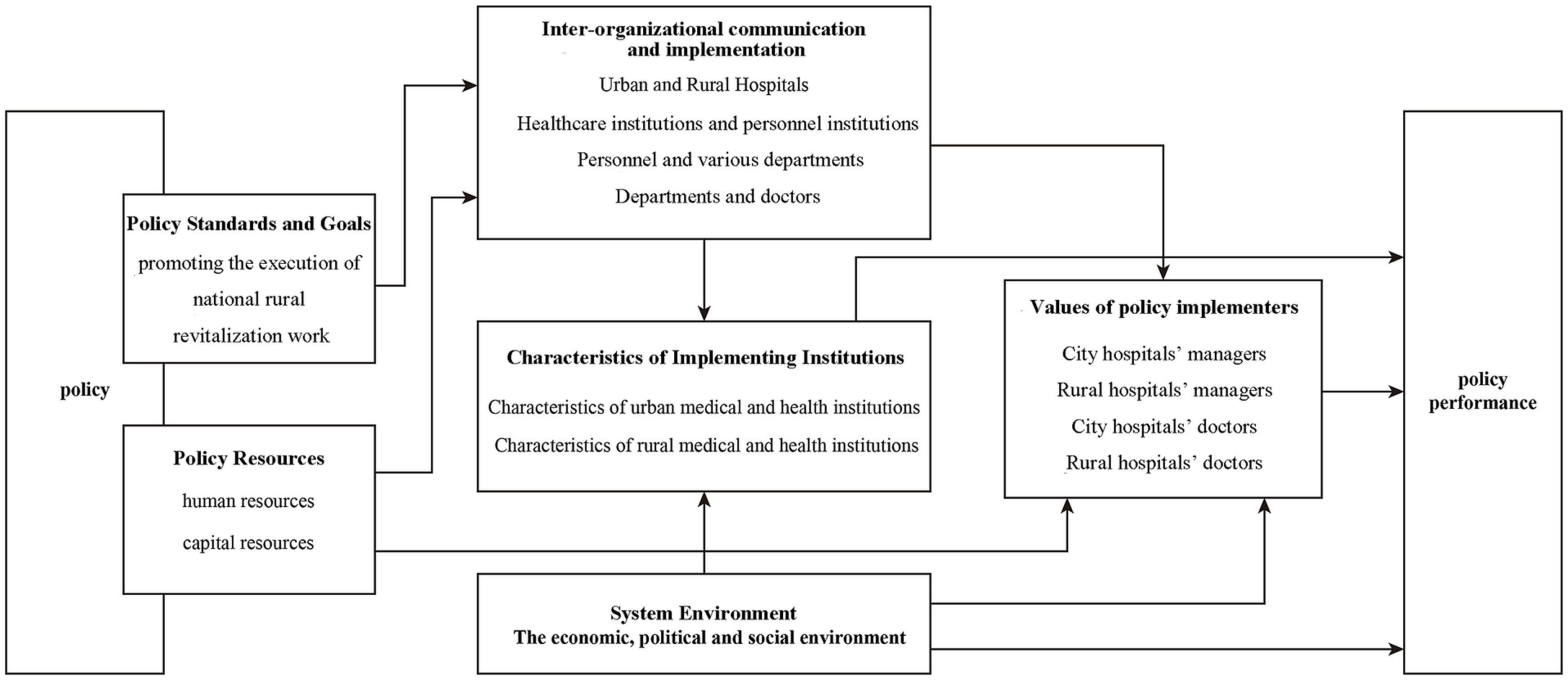
Analytical framework of UDSR policy in Beijing. This framework was developed by the authors building upon Van Meter and Van Horn’s policy implementation model, incorporating the characteristics of UDSR policy and the research context.

With “urban doctors serving rural areas” as the focal policy implementation process, this study adopts the Horn-Mitt policy implementation model as a theoretical analysis framework. This framework serves as a guiding framework for the theoretical analysis, as shown in Figure 1.

## 3 Data and method

### 3.1 Case selection

Against the backdrop of China’s efforts to bridge urban–rural healthcare disparities, the UDSR policy in Beijing has evolved as a targeted intervention rooted in both national mandates and local governance needs. In 2006, the Beijing Municipal Health Bureau and the Municipal Personnel Bureau jointly issued a directive titled “Notice on Relevant Issues Concerning Urban Doctors Serving at Grassroots Medical Institutions before Being Promoted to Professional and Technical Positions (44). “This policy strictly regulates the tenure of urban doctors’ service in remote towns and townships. It stipulates that urban doctors must complete service for a whole year in rural grassroots medical institutions-particularly in remote suburban districts before eligibility for promotions to senior professional and technical positions. This policy design, aligning with the central government’s directive on ‘counterpart support’ for rural health work, aims to address structural imbalances: urban hospitals in Beijing, boasting advanced medical resources and expertise, are tasked with alleviating shortages in rural areas characterized by limited access to quality care, insufficient specialist coverage, and weaker emergency response capabilities. By 2017–2019, the policy had matured into a systematic practice involving annual deployment quotas, standardized evaluation metrics, and multi-source funding mechanisms, which lay the groundwork for this study’s case selection and analysis.

The case selection in this study adheres to the following criteria:

1. Typicality of the case. Cases were selected where the supporting hospital is located in the core urban area of Beijing, while the recipient rural hospital is located in the outskirts. These cases represent common practices and prevalent issues encountered in the implementation of the UDSR policy. These cases represent common practices and prevalent issues encountered in the implementation of the UDSR policy, as the selected supporting hospitals are public medical institutions affiliated to the Beijing Municipal or District Health Commission. They are mandated by policy to undertake urban–rural counterpart support tasks, reflecting the typical characteristics of top-down policy execution in China’s public healthcare system.
2. Heterogeneity of the case. Given the disparity in available medical resources and political leverage among hospitals of different levels, cases from hospitals at varying levels were included in the selection process. The dataset includes a total of 13 hospitals, with 6 Level-2 hospitals and 7 Level-3 hospitals: Level-3 hospitals are generally larger in scale with more advanced equipment and richer specialist resources, while Level-2 hospitals, though smaller, have relatively comprehensive departments and closer connections with grassroots institutions. These samples could capture differences in policy execution capacity across hospital tiers.
3. Scientific and continuous data. To mitigate the influence of unforeseen public health crises like the COVID-19 pandemic in 2020 on the analysis results, data spanning three consecutive years (2017–2019) were collected. In total, 39 case samples were obtained over the three-year period. We adopt a purposive sampling approach. The inclusion criteria focused on hospitals that continuously participated in the UDSR policy during 2017–2019 and maintained complete records. Hospitals with discontinuous participation or incomplete data were excluded. This sample size is determined by the total number of eligible hospitals in District Xicheng that meet the criteria, ensuring full coverage of policy-implementing entities in the study area while satisfying the requirements of fsQCA for small-sample configurational analysis. The characteristics of the sample hospitals are presented in Table 1.

**Table 1:** Hospital characteristics of counterpart support hospitals.

#	Hospital name	Level	Governing body	Established	Gross floor area (m^2^)	Bed size	Staff size (person)	Population served (per day)
1	H1	Tertiary	Beijing municipal health commission	1981	42,097	1700	2,396	13,500
2	H2	Tertiary	Beijing municipal health commission	1947	25,600	300	482	2000
3	H3	Secondary	Xicheng district health commission	1960	12,000	185	480	1,000
4	H4	Tertiary	Beijing municipal health commission	1937	26,800	400	1,187	2000
5	H5	Tertiary	Beijing municipal health commission	1950	106,500	710	1,557	1700
6	H6	Secondary	Xicheng district health commission	1945	24,287	286	388	1,200
7	H7	Secondary	Xicheng district health commission	1960	5,688	100	416	1,650
8	H8	Secondary	Xicheng district health commission	1974	11,000	52	205	934
9	H9	Tertiary	Beijing municipal health commission	1906	18,563	30	600	1700
10	H10	Secondary	Xicheng district health commission	1949	10,341	350	351	417
11	H11	Secondary	Xicheng district health commission	1958	3,000	300	130	1,300
12	H12	Tertiary	Beijing municipal health commission	1953	32,000	457	915	1,650
13	H13	Tertiary	Beijing municipal health commission	1958	157,000	1,403	3,571	4,200

To protect hospital confidentiality, hospital names have been replaced with H* codes.

### 3.2 Data collection

The data in this study comes from two sources: Firstly, they come from the “Statistical Form of Workload for Urban and Rural Counterpart Support in Beijing,” issued by the Beijing Municipal Health Commission and filled out by hospitals; Secondly, adhering to the research theoretical framework, data collection was conducted based on a logical structure. This structure covers policy standards and goals, policy resources, implementation methods, characteristics of implementing institutions, and value orientations of implementers. Environmental factors in the theoretical framework, like the level of media attention to the policy and the beliefs and attitudes of doctors and patients in urban and rural hospitals, are subjective and hard to measure. Therefore, these factors were not included in the questionnaire survey. The questionnaire comprises various types of questions, including basic information questions, multiple-choice questions, fill-in-the-blank questions, multiple-response questions, and subjective questions regarding policy cognition. Additionally, validation questions were designed for subjective questions to ensure effective content acquisition. Details of the questionnaire are shown in the Appendix.

### 3.3 Research methods

The implementation of UDSR policy is a complex process influenced by multiple factors, and the policy effectiveness results from the interplay of these factors. To comprehensively analyze causal conditions and outcome, this study incorporates the qualitative comparative analysis (QCA) method.

#### 3.3.1 Method selection

The QCA method is selected by the following considerations: Firstly, this method could address complex causality. Policy effectiveness in UDSR is determined by configurations of variables rather than single factors, which aligns with our research question of uncovering implementation pathways instead of isolated factors. Secondly, it suits for small sample sizes with multi-level data. Our sample is typical of case studies focusing on Beijing’s specific policy. The QCA is superior to quantitative methods for small samples, as it avoids issues of statistical power and instead emphasizes configurational patterns (51). Thirdly, it could capture the dynamic trajectories. By integrating time-series QCA (52), fsQCA overcomes the “temporal blind spot” of traditional static methods. This allows us to trace evolutionary trajectories, which is central to understanding how policy effectiveness evolves over time. Fourthly, the fsQCA could handle ordinal outcomes. Unlike crisp-set QCA limited to binary outcomes, fsQCA accommodates the ordinal nature of our dependent variable. This flexibility ensures nuanced analysis of varying effectiveness levels. Moreover, considering the limited number of secondary and tertiary hospitals participating in the paired support work in X district of Beijing, we simplified the analysis using QCA software by setting the consistency threshold to 0.8.

Following the selection of the outcome variable, the configuration analysis yields three types of solutions: complex solutions, intermediate solutions, and simple solutions. The intermediate solution with reasonable evidence and moderate complexity is usually the first choice for reporting and interpretation in QCA research (42). Therefore, this study selects the intermediate solution to explain the implementation effectiveness of the UDSR policy. By reporting the intermediate solutions and integrating them with the simple solutions, we could effectively identify core and marginal conditions. Boolean minimization techniques were applied to obtain the configuration outcomes. Utilizing Fiss’s (53) methodological framework for classifying conditions, we distinguish between core and marginal conditions. The preceding conditions that simultaneously appear in the parsimonious solution and intermediate solution are defined as core conditions, while the conditions that appear in the intermediate solution but are excluded in the parsimonious solution are defined as marginal conditions.

This approach provided a nuanced understanding of the factors influencing policy outcomes. All the data were encoded and analyzed using the QCA software. The 75, 50, and 25% quantiles were employed to represent belonging point, crossover point, and non-belonging point, respectively, and were transformed into fuzzy set membership scores.

#### 3.3.2 Variable coding

To ensure clarity in translating abstract policy concepts into analyzable data, this section follows a three-step logic: conceptualizing key variables to selecting measurable indicators, and to defining coding rules. This process systematically bridges theoretical ideas with empirical analysis, aligning with the UDSR policy context.

##### 3.3.2.1 Step 1 Conceptualization of key variables

First, we clarify the abstract policy—related concepts and their relevance to the UDSR policy goals, thereby laying the foundation for measurable indicators.

Policy Effectiveness: It is operationalized as “rural medical technology improvement,” focusing on cumulative educational and service-oriented inputs like training and consultations from urban to rural hospitals. This reflects the policy’s goal of enhancing rural medical capacity.

Policy Implementation Dimensions: These dimensions include policy standards, policy resources, implementation mode, implementers’ features, and value orientation. Each dimension is based on theoretical analysis and practical policy execution logic.

##### 3.3.2.2 Step 2 Indicator selection: translating concepts to measures

Next, we translate abstract concepts into observable indicators, ensuring alignment with both policy logic and research goals, and that data can be obtained from administrative records and surveys.

Policy Standards: The number of counterpart support projects, which reflect how policy requirements are transformed into actionable tasks.

Policy Resources: The diversification of subsidy sources, measuring funding accessibility as multi-source subsidies often signal stronger resource support for rural hospitals.

Implementation Mode: Smoothness of communication, capturing stakeholder coordination efficiency—a critical factor in avoiding policy execution delays.

Implementers’ Features: Expertise matching and Prescription right, where “expertise matching” reflects professional alignment with rural needs and “prescription right” measures decision-making power in rural settings.

Value Orientation: Policy awareness of stakeholders, reflecting cognitive and attitudinal alignment with policy goals.

##### 3.3.2.3 Step 3 Variable coding: operationalizing measures into data

Finally, we specify how indicators were coded into analyzable data to ensure consistency and compatibility with fsQCA. The outcome variable was operationalized as a cumulative score of technical inputs, such as training sessions and consultations, with raw counts standardized to a 0–1 fuzzy-set scale for configurational analysis. Explanatory variables were coded according to their conceptual meanings: categorical variables, such as communication smoothness and prescription right, were binary-coded; ordinal variables, such as expertise matching and policy awareness, were scored on Likert-like scales (1–5) and calibrated for fsQCA; continuous variables, such as the number of counterpart support projects, were grouped into ordinal categories based on theoretical thresholds. Detailed coding rules for these specific indicators are presented in Table 2.

**Table 2:** Variable setting and coding based on policy implementation model.

Variables of policy implementation model	Evaluating indicator	Measurement of variables
Outcome variable	Improvement of rural medical technology	The total count encompasses a range of educational and operational metrics, including: Training sessions focused on diagnostic and treatment technologies. Instances of outpatient diagnostic and treatment teachings. Surgical procedure demonstrations conducted. Consultations held for complex and challenging medical conditions. Teaching rounds facilitated to enhance clinical knowledge. Academic lectures delivered to broaden medical understanding. Workshops and trainings provided by professional business trainers. These cumulative figures reflect the comprehensive educational efforts and service capacities of the medical institution in question.
Policy standards and objectives	Number of counterpart support projects	Accumulation of the number of counterpart support projects provided by urban medical institutions.
Policy resources	Diversification of subsidy sources	If both the superior department and the urban hospital pay the subsidy, it is recorded as 1. If only one of the departments pays the subsidy, it is recorded as 0.5 If neither department pays the subsidy, it is recorded as 0.
Mode of implementation	Whether the communication in the policy implementation process is smooth	Smooth communication channels, indicating effective and unhindered communication between relevant stakeholders, is recorded as 1. Otherwise, it is recorded as 0.
Implementers’ features	The expertise of urban doctors matching the needs of rural hospitals	The count of individuals without counterparts is 1, while a handful of professional counterparts are rated as 2. Some professional counterparts are rated as 3, the majority of professional counterparts are rated as 4, and all professional counterparts are rated as 5.
	Urban doctors’ prescription right in rural hospital	When urban doctors have prescription rights, it is recorded as 1; otherwise, it is recorded as 0.
Value orientation	Hospital department managers ‘awareness of the policy	Based on the subjective question and the validation question, the degree of cognition is scored, with 0 indicating low policy awareness and attitude, and increasing values reflecting higher levels of policy cognition and attitude.

### 3.4 Ethical approval

Although Chinese regulations stipulate that research without biological experimentation does not necessitate ethical review (54, 55), we still submitted our study for ethical approval and obtained it. The research design, including the interview protocol and questionnaire content, received ethical approval (Approval No.: ZYLL 2022-019) from the Ethics Committee of Beijing Xicheng District Zhanlan Road Hospital on 8 October 2022. All participants, including managers and doctors from urban and rural counterpart support hospitals, provided written informed consent prior to their involvement in interviews and questionnaire surveys, confirming their voluntary participation and agreement to the use of data for research purposes. We conducted data collection and recording strictly in line with the approved protocol, ensuring the protection of participants’ privacy and rights.

## 4 Results

The analysis focused on examining the necessity of individual conditions and the sufficiency of condition combinations.

### 4.1 Necessity test of single factor

We conducted a univariate analysis to determine the necessary condition for all variables, and the results are shown in Table 3. According to Ragin’s criterion (56), where a consistency value exceeding 0.9 is required for a variable to be considered a necessary condition for an outcome, none of the six individual variables exhibit consistency above this threshold. This indicates that no single variable alone could fully explain the progress of the advancement of rural medical technology. However, the consistency values for the number of support projects, the number of subsidy funding departments, and the prescription authority of doctors deployed to rural areas exceeded the threshold of 0.8.

**Table 3:** Necessity analysis of individual condition variable.

Variable	Consistency	Coverage	Variable	Consistency	Coverage
Number of support projects	0.817	0.803	~Number of support projects	0.294	0.288
Professional matching	0.629	0.614	~Professional matching	0.496	0.490
Policy comprehension	0.782	0.802	~Policy comprehension	0.325	0.306
Diversification of subsidy sources	0.864	0.580	~Diversification of subsidy sources	0.194	0.354
Communication channel	0.769	0.566	~Communication channel	0.231	0.339
Prescription right	0.825	0.607	~Prescription right	0.175	0.258

The symbol “~” stands for “absence of”.

### 4.2 Sufficiency analysis of condition combinations

Table 4 reports the result of the conditions necessary for the effectiveness of the UDSR policy. It also highlights the significance of condition combinations and the need for further research. The overall consistency score was 0.972, exceeding the threshold of 0.75. This result indicates a strong alignment between the identified condition configurations and the effectiveness of the policy. This finding suggests that these configurations are likely necessary conditions to achieve the policy’s outcomes. The overall coverage rate indicates that about 68.2% of the policy’s effectiveness can be explained by these configurations. “Number of Support Projects,” “Professional Matching,” “Diversification of Subsidy Sources,” and “Prescription Rights” appear in multiple configurations. This result suggests that they may be core conditions for achieving policy effectiveness.

**Table 4:** The condition configuration of the effect of the UDSR policy.

Conditional configuration	1	2	3	4	5
Number of support projects	●	●	⊗	⊗	●
Professional matching	●	●	⊗	●	⊗
Policy comprehension	●		●	⊗	●
Diversification of subsidy sources	●	●	⊗	⊗	●
Communication channel		•	⊗	•	⊗
Prescription right	⊗	●	●	●	●
Original coverage	0.127	0.416	0.054	0.052	0.132
Unique coverage	0.127	0.317	0.054	0.052	0.033
Overall consistency	0.972				
Overall coverage	0.682				

‘●’ Indicates that the core condition exists, ‘•’ indicates that the edge condition exists, ‘⊗’ indicates that the core causal condition is missing, and the condition does not exist, and ‘blank’ indicates that the condition may or may not appear in the configuration (61, 62).

“Communication Channel” only appears in configurations 3 and 4, while “Policy Comprehension” exists in configurations 2 and 5. This indicates they may be peripheral conditions, influencing policy outcomes but perhaps not being the primary drivers. Configuration 2 has the highest original coverage (0.416), indicating it is the most common configuration. However, it also has the highest unique coverage (0.317). This high unique coverage suggests that this combination alone can explain a substantial portion of the policy’s effectiveness. No single condition can alone explain most of the policy’s effectiveness. This fact suggests that combinations of conditions play a vital role in achieving the desired outcomes of the policy. Although some conditions appear in multiple configurations, additional qualitative or quantitative research may be required. Such research can help us better understand how these conditions interact and specifically affect policy effectiveness.

### 4.3 Implementation pathway of factors influencing USDR policy effectiveness

By analyzing conditional configurations and integrating theoretical research, the mechanism of the UDSR policy can be summarized into four distinct pathways.

Pathway 1 is Goal and Cognition-driven. Configurations 1 and 2 highlight the significance of ample project support, clear policy comprehension, and efficient communication channels as core variables. While professional matching in Configuration 2 influences technological investment, it is not a central factor. The presence of multiple subsidy sources and clear prescription rights are important, but they do not need to occur simultaneously. This pathway emphasizes the proactive engagement of urban hospital managers and doctors in policy execution, thus being labeled as Goal and Cognition-driven.

Pathway 2 is Professional Matching-driven. Since both Configurations 2 and 4 underscore the importance of prescription rights for urban doctors working in rural hospital, which align with professional matching. Increased awareness significantly promotes policy implementation. Although Configuration 2 shows greater hospital support than Configuration 4, the latter is more universally applicable, making it the Professional Matching-Driven pathway.

Pathway 3 is External Funding-driven. Prescription rights are a constant core variable across Configurations 2, 3, 4, and 5. Configurations 3 and 5, however, prioritize the number of subsidy-granting departments, a central factor in these setups. The Van Horn and Van Meter model indicates that the diversity of subsidy sources signifies policy resource investment, particularly in covering rural doctor costs. As this pathway centers on the scale of external funding, it is categorized as External Funding-Driven.

**Figure 2:**
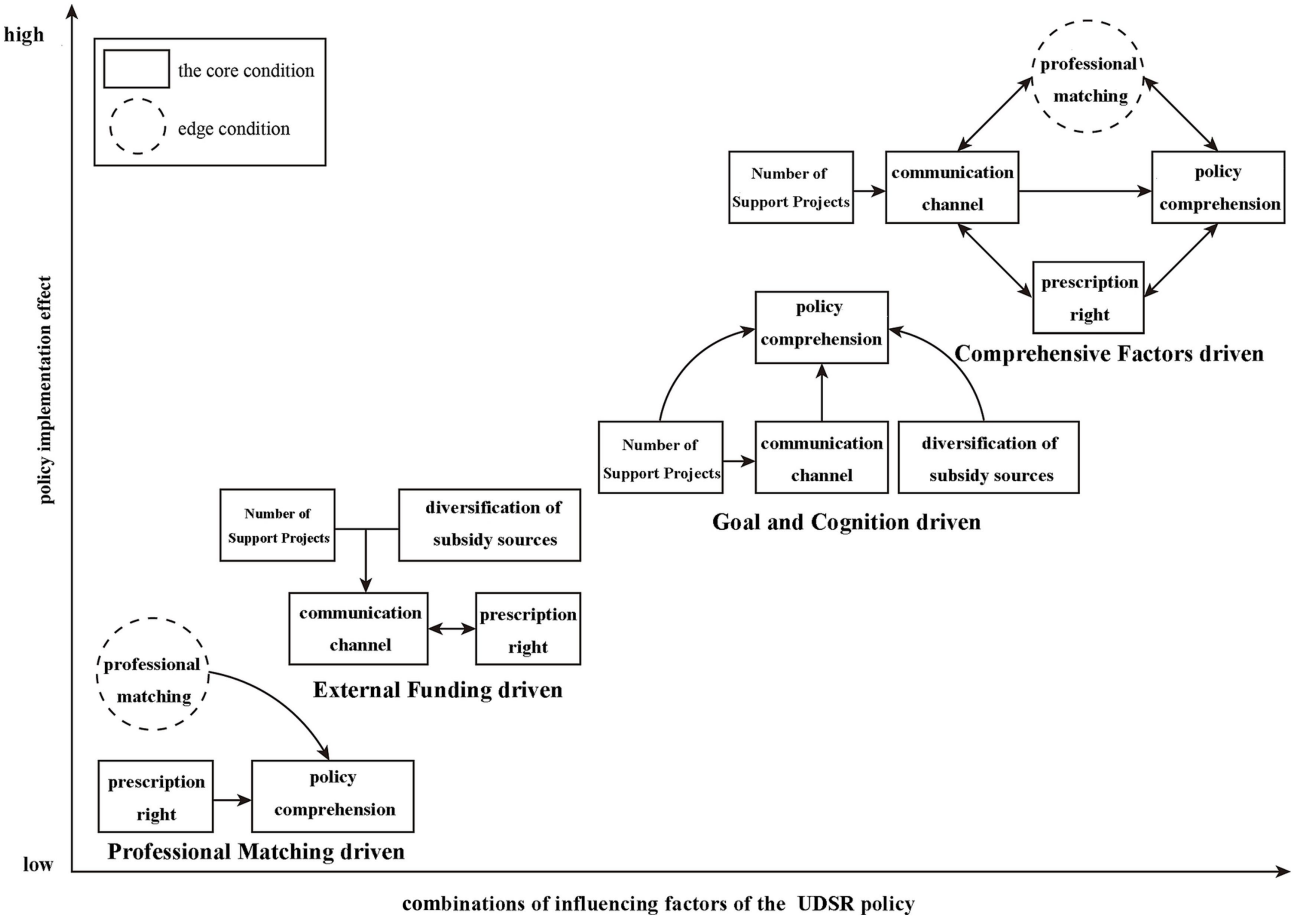
Influencing factors and implementation path of the UDSR policy in Beijing.

Pathway 4 is Comprehensive Factors-driven. Configuration 2 is notable because it includes project support, prescription rights, communication channels, subsidy source, and professional matching as core or marginal variables. It embodies the synergistic effect of multiple factors. With core conditions such of subsidy sources, policy awareness, project support, communication, and prescription rights, Configuration 2 plays a crucial role in advancing the policy’s rural service objectives. The marginal condition of professional matching also has an impact. This pathway demonstrates the highest adaptability and stability, making it the most effective in facilitating the policy’s successful implementation (see Figure 2).

### 4.4 Robustness test

This study employed two methods to test the robustness of the results. First, the analysis was refined by increasing the consistency threshold to 0.85. This adjustment simplified the process and revealed that the configurations of technical and non-technical inputs in the experimental group were completely consistent.

Second, due to the causal asymmetry principle in qualitative comparative analysis, the conditions for high technical input cannot explain the occurrence of low technical input. To address this, the study conducted a directional test on the outcome variable, and this test yielded reverse analytical results. Therefore, the configuration results have been proven to be robust, as they have withstood examination through both enhanced methodological rigor and directional testing.

### 4.5 Multi-period comparative analysis

This paper delves deeper into the dynamics of factor configurations and their evolution impacting the UDSR policy by conducting a comparative analysis that spans from 2017 to 2019. Using standardized calibration criteria and percentile calibration benchmarks, this study presents a time-segmented analysis of the UDSR policy configuration. The result showed that there were three configurations for each year, and culminating in a total of nine condition configurations across the three-year periods. Each individual condition configuration, as well as the overall consistency for each time period, surpassed the 0.75 threshold. The overall coverage rates are 0.672, 0.709, and 0.541 for the respective periods, shown in Table 5.

**Table 5:** The condition configuration of the effect of the UDSR policy in multiple periods.

Conditional configuration	2017			2018			2019		
	Configuration 1	Configuration 2	Configuration 3	Configuration 4	Configuration 5	Configuration 6	Configuration 7	Configuration 8	Configuration 9
	All-factor-driven type	All-factor-driven type	External capital-driven	Practice matching driven type	All-factor-driven type	External capital-driven	Practice matching driven type	Goal and cognitive driven	All-factor-driven type
Number of Support Projects	●	●	⊗	⊗	●	●	⊗	●	●
Professional matching	●	●	⊗	●	●	⊗	●	●	●
Policy comprehension	●		●		●	●	○	•	•
Diversification of subsidy sources	●	●	⊗	⊗	●	●	⊗	●	●
Communication channel	●	●	⊗	●	●	⊗	●	⊗	●
Prescription right		●	●	•		•	●	⊗	●
Original coverage	0.966	0.965	1	1	0.973	0.991	1	1	0.995
Unique coverage	0.507	0.500	0.090	0.090	0.530	0.190	0.079	0.157	0.305
Overall consistency	0.083	0.075	0.090	0.090	0.429	0.089	0.079	0.157	0.305
Overall coverage	0.974			0.977			0.997		
Number of support projects	0.672			0.709			0.541		

**‘**●’ Indicates that the core condition exists, ‘•’ indicates that the edge condition exists, ‘⊗’ indicates that the core causal condition is missing, the condition does not exist, ‘○’ indicates that the edge causal condition is missing, and ‘blank’ indicates that the condition may or may not appear in the configuration (61, 62).

In examining the evolution of the condition configurations affecting policy effectiveness over multiple time periods, this study identifies two trajectories: the “transitional trajectory” and the “dominant trajectory,” according to Litrico’s framework (43). The configuration driven by comprehensive factors appeared consistently across all three periods and had a significant impact on the UDSR policy effectiveness. This is characterized as the dominant trajectory. Conversely, the external funding-driven configuration was present in 2017 and 2018, but absent in 2019. The practice match-driven configuration emerged in both 2018 and 2019, yet was not detected in 2017. The goal and cognitive-driven configuration only appeared in 2019, indicating a clear transitional trajectory.

## 5 Discussion

### 5.1 Findings interpretation and comparison

Our fsQCA results reveal four equifinal pathways to high UDSR policy effectiveness. None of the six individual conditions meets Ragin’s 0.9 threshold for necessity, confirming that the improvement of rural medical service is driven by configurational causation rather than any single factor. This result is consistent with recent studies on multi-level policy implementation (27, 57). However, three variables—the number of support projects, the diversification of subsidy sources, and urban doctors’ prescription rights—achieved consistencies of 0.8, indicating that they are core determinants. This finding is partly echoed in other counterpart-support studies (35, 39, 58).

Pathway 1 (Goal-and-Cognition-driven) shows that high project volume, high policy comprehension, and smooth communication can generate substantial technological inputs, even when professional matching is only marginal. This echoes Van Meter & Van Horn’s proposition that clear standards and inter-agency communication are decisive in hierarchical, top-down programmes (57). Pathway 2 (Professional-Matching-driven) indicates that clinical autonomy and skill compatibility are more important than financial incentives alone. However, these factors and pathway has been overlooked in previous studies (23, 26). Pathway 3 (External-Funding-driven) illustrate a resource-first logic, which is often highlighted in the early-stage counterpart support studies (38, 59). Yet its low coverage suggests indicates that this pathway is context-dependent and less sustainable when external funding decreases. Pathway 4 (Comprehensive-Factors-driven) integrates all five enabling conditions and achieves the highest unique coverage. This finding is consistent with studies suggesting that integrated interventions are essential for sustainable improvements in rural healthcare systems (40).

The dominant trajectory across 2017–2019 is Pathway 4, underscoring that holistic governance becomes institutionalized over time. Conversely, the transitional trajectory—shifting from the external funding path to the professional matching path and finally to the goal & cognition path—demonstrates adaptive policy learning. External funding initiates the process. As the novelty of fiscal support diminishes, attention shifts to internal coordination, confirming Litrico’s argument that the interpretation of issues evolves with stakeholder framing and resource maturity (60).

### 5.2 Research contributions

This study makes significant theoretical contributions. First, it unveils complex causal configurations beyond single-factor analysis. Existing literature often focus on empirical summaries and single-case analyses with limited exploration of the specific factors and the synergistic effects of multiple factors. In contrast, this study employs fsQCA and identifies three critical factors and their configurational effects, advancing the understanding of multiple causes for a single outcome in policy implementation. Second, it deepens the theoretical understanding of policy implementation pathways of the counterpart support policy. While previous research have discussed medical counterpart support from perspectives of policy tools or evaluation frameworks, this study adopts the Van Meter–Van Horn policy implementation framework to structure the analysis of influencing factors. This not only validates the framework’s applicability in medical counterpart support policies but also provides a theoretical basis for understanding how multiple factors interact to shape policy outcomes, filling the gap in theoretical-driven pathway analysis. Third, it reveals evolutionary trajectories of policy effectiveness over time. In contrast of existing literature that rarely explores the changes in influencing factors across different periods, this study identifies two evolutionary trajectories, offering a nuanced understanding of the adaptive evolution of policy effectiveness.

In terms of practical implications, first, the identification of critical factors and their configurations provides practical guidance for policy-makers. They can focus on these key elements when formulating and adjusting counterpart support policies to improve policy effectiveness. Second, validating the Van Meter-Van Horn framework’s applicability in this context offers a practical analytical tool for future policy research and implementation. It helps practitioners systematically analyze and address issues in medical counterpart support. Third, the disclosure of the evolutionary trajectories of policy effectiveness can assist in long-term policy optimization. By understanding how policies adapt over time, stakeholders can make more informed decisions to ensure the sustainable development of counterpart support policies, providing valuable insights for the long-term optimization of these policies in practice.

### 5.3 Limitations and future research

Despite the contributions, this study has several limitations. Firstly, the sample is restricted to public hospitals in Beijing. Given the specific policy context, the generalizability of the findings may be limited. As a result, the identified pathways and trajectories may not fully apply to other provinces or rural areas with less developed medical infrastructure. Secondly, although the study identifies critical factors such as support projects and prescription rights, the fsQCA method primarily focuses on configurational effects. It does not quantify the marginal impact of individual variables. This limits the ability to determine the precise weight of each factor in driving policy effectiveness, leaving room for further exploration of causal mechanisms between specific variables. Thirdly, due to measurement challenges, environmental factors in the Van Meter–Van Horn framework—such as media attention and doctors’/patients’ socio-psychological attitudes—were not included in the analysis. These unobserved factors may interact with the included variables to shape implementation outcomes. Their omission means that some subtle dynamics in policy execution might have been overlooked.

Future research could address these limitations by: (1) Expanding the sample to include hospitals in other regions, such as western China or small cities. This would facilitate cross-regional comparisons and test the adaptability of the identified policy implementation pathways; (2) Integrating fsQCA with quantitative methods. This integrated approach can quantify the relative importance of key factors while still maintaining insights into factor configurations; (3) Developing more refined measurement tools for environmental and socio-psychological variables. Researchers can explore how these factors interact with observed factors, thereby enhancing the comprehensiveness of the analytical framework.

## 6 Conclusions and policy implications

### 6.1 Conclusion

This study investigates the urban–rural medical counterpart support policy, which aims to reduce disparities in medical service quality. The samples are collected from Beijing public hospitals, and the analysis is guided by the Horn-Mitt policy implementation framework. To explore the factors influencing policy effectiveness and operational pathways, this study uses interview questionnaires combined with fsQCA. The findings indicate that no single factor alone is a necessary condition for improving rural medical services. However, some key elements are very important. These elements include the number of support projects, diversified subsidies, and doctors’ prescription rights play critical roles. Four operational pathways were discovered. They are the goal-cognition-driven pathway, professional matching-driven pathway, external funding-driven pathway, and comprehensive factor-driven pathway. Among them, the comprehensive factor-driven pathway is the most effective. Through multi-period analysis, two types of trajectories were revealed. One is the dominant trajectory, which is characterized by continuous comprehensive factor configurations. The other is the transitional trajectories, which change from relying on external funding to achieving professional alignment. These results prove that the Horn-Mitt framework could be applied in this context. They also make clear how different factors work together during policy implementation. In addition, the results provide practical insights for improving counterpart support strategies to enhance rural medical services.

### 6.2 Policy recommendations

Based on our empirical findings and the Horn-Mitt policy implementation framework, the following targeted recommendations are proposed to assist governments and hospitals in addressing urban–rural healthcare disparities.

For municipal policymakers, it is important to ensure the systemic coordination based on the finding that multi-factor synergies are most effective, aligning with the Horn-Mitt framework. It is recommended to design phased strategies to guide the transition from external funding dependence to comprehensive collaboration. The standardized goals linked to the support projects is needed. For local health commissions, we firstly propose to establish regular cross-institutional communication mechanisms. These mechanisms can help improve cooperation and information sharing among different medical institutions. The second is to prioritize resource allocation. Local health commissions should focus on key areas, like diversifying subsidy sources and clarifying prescription rights to strengthen professional matching, drawing on the critical role of communication channels and inter-organizational cooperation. For urban public hospitals, it is crucial to refine talent deployment to align with rural needs and expand high-quality support projects while ensuring doctors’ prescription rights, reinforcing the goal-cognition pathway. This recommendation is grounded in the significance of support projects and professional matching in effective pathways. For rural recipient hospitals, it is advisable to proactively communicate service gaps to urban partners and strategically utilize subsidies to maximize the impact of urban medical resources on rural healthcare, thereby promoting rural medical development.

## Data Availability

The original contributions presented in the study are included in the article/Supplementary material, further inquiries can be directed to the corresponding author.
