# Supplementary material for: Policy practice for narrowing urban–rural healthcare gaps: determinants and implementation path of the urban doctors servicing rural areas policy in Beijing, China
Source: Front Public Health. 2025 Sep 22;13:1456142. doi: 10.3389/fpubh.2025.1456142 (PMC12497779; doi:10.3389/fpubh.2025.1456142)
Supplement: Supplementary file 1 [file Table_1.DOC]

**Appendix Survey content for city hospital management departments**

Analysis of Factors Affecting the Implementation of UDRS in X District: A Questionnaire Survey.

This questionnaire involves data from 2017 to 2019, with the same questions each year. In order to obtain effective data to address the issues in the service of urban doctors going to rural areas, please fill out according to your actual situation, without including unit names, not disclose data. Thank you for your cooperation.

1. Hospital Level [Single Choice Question]*

○ Level two

○ Level Three

The following questions are based on the annual data and actual situation from January to December 2017.

The content of the specific questionnaire remains the same for the years 2017, 2018, and 2019. Only the content for 2017 is displayed.

2. Forms of cooperation carried out in the urban-rural paired assistance (limited to the pairing assistance between Beijing urban hospitals and medical institutions in Beijing's rural areas).

□Outpatient treatment

□Receive further education.

□Perform surgery (lead surgeon)

□Surgical teaching (education)

□Difficult disease consultation.

□Teaching rounds

□Physical examination for health.

□Academic Lecture

□Departmental business training.

□Free Medical Consultation Event

□Establishing specialized departments.

□Donation of money and goods

3. When providing outpatient diagnosis and treatment for local patients, do doctors who have gone to the countryside within the year have the prescription authority (the "going to the countryside" program is only for city hospitals in Beijing to assist rural medical institutions in Beijing), if they do not have prescription authority, please specify the reason. [Single choice question]

○ yes.

○ No ________________*

Dependent on the first option of the second question.

4. Is outpatient teaching conducted?

○ yes.

○ No

Dependent on Option 1 of Question 2.

Annual outpatient teaching quantity within the year [Fill in the blank]*

_________________

Dependent on the 1st option of the 4th question.

6. Number of teaching surgeries conducted annually [Fill in the blank]*

_________________

Dependent on the 4th option of question 2.

7. Number of difficult disease consultations completed during the year (cumulative remote on-site) [Fill in the blank]*

_________________

Depending on the 5th option in question 2.

8. Number of teaching rounds completed within the year.

_________________

Dependent on the 6th option in question 2.

9. Number of academic lectures completed during the year (excluding internal departmental lectures)[Fill in the blank]*

_________________

Dependent on question 2, option 8.

10. Number of business training sessions completed within the year (within the department) [Fill in the blank]*

_________________

Depending on the 9th option of the 2nd question.

11. Number of featured specialties established within the year [Fill in the blank]*

_________________

Dependent on the 11th option of question 2.

12. Annual donation and donation-in-kind equivalent amount (unit: RMB) [Fill in the blank]*

_________________

Dependent on option 12 of question 2.

13. Has any allowance been issued to the personal account of urban doctors?

○ Only government subsidies (subsidies from any superior department)

○ Only unit subsidy.

○ Both government subsidies and corporate subsidies exist.

○ None

14. Which level of staff from the city hospital visits rural medical institutions for communication and signing of the annual agreement? [Multiple choice]*

○ City hospital leader

○ Deputy Director of City Hospital

○ Head of Department in City Hospital

○ City hospital delegates doctors to sign on behalf in rural areas.

○ Other____________

15. Are there any needs to send personnel who have applied to go to the paired assistance but are unable to allow them to participate in going to the countryside within the year? [Single choice question]

○ yes.

○ No

16. Healthcare professionals who registered for the program wanted to go to the countryside, but □ the hospital could not arrange their trip for the following reasons [Multiple choice]*

□ The major does not match, and the other party does not require.

□ The department has too many applicants in the same year and needs to ensure the operation requirements of the department.

□ The number of registered unit personnel for the year is too high to arrange.

□ The registrants have no promotion requirements.

□The applicants have the need for promotion but do not meet the promotion requirements, so they are not currently being considered.

□ Others ________________*

Dependent on Option 1 of Question 15.

17. What is the approximate difference between the number of registered personnel and the number of personnel actually arranged to go to the countryside in the current year? [Fill in the blank]*

_________________

Dependent on the 1st option of question 15.

18. Number of dispatched doctors annually (actual number of doctors sent to rural healthcare institutions in Beijing) [Fill in the blank]

_________________

1. Is the professional department where the dispatched doctors are sent to rural medical institutions in line with their expertise? [Single choice question]*

○ There is no counterpart personnel.

○ Few professional counterparts.

○ Part of the major is matched.

○ Most are professional and relevant.

○ All are matched.

20. How many times have there been communications between the implementation of the "City Doctor Serving the Rural Area" policy and rural medical institutions this year?

○ 0 times

○ One time to ten times

○ 10 times (excluding 10 times) - 30 times

○ Between 30 times (exclusive) to 50 times.

○ More than 50 times (excluding 50 times)

21. What aspects are included in the communication with rural medical institutions this year? [Multiple choice]*

□ Arrangement and identification of personnel going to the countryside.

□ Professional knowledge, technical guidance issues

□ Device facilities and other hardware conditions.

□ Actual working conditions of rural migrant workers

□ Cooperation with urban hospitals.

□ Rural healthcare institution demand.

□ The summary of work tasks required by the superior department, communication related to data reporting, etc.

□Other ___________________

22. Has there been any contradiction, disharmony, or lack of smoothness in the implementation process of the annual policy of sending urban doctors to the countryside (Those who strongly request to participate but are unable to participate and are dissatisfied with the arrangement for countryside service should also be considered as not smooth)? [Single choice question]

○ yes.

○ No

1. What are the aspects of the disharmonious factors that have appeared? [Multiple choice]*

□ In the clinical department.

□ Between clinical departments and responsible departments.

□ Between the clinical department and the human resources department

□ The relationship between the department head and the human resources department.

□ Between hospitals and superior departments (including superior supervisory departments, superior personnel departments, etc.)

□ Hospitals and their paired-assistance units.

Dependent on the first option in question 22.

(2018, 2019 content is the same as above)

To better understand the needs of various units and improve policies, this question will no longer consider annual situations. It is necessary to evaluate policies based on actual circumstances. Please fill out the answer carefully.

62. What is the requirement for urban doctors to go to the countryside and the number of days they must serve before being promoted under the personnel promotion policy of "urban doctors serving in rural areas"? [Multiple choice question]*

○ Great requirement, unit personnel have no complaints, keep it up.

○ Good requirements, no complaints from the staff, can be maintained.

○ Generally, there are some complaints from unit personnel, which can be maintained after modification.

○ The requirement is not very good, and there are many complaints from the staff, so it needs a major overhaul.

○ It's a very bad request, the unit personnel have been resentful for a long time, so I suggest cancelling it.

63. As the head of the department in charge, do you find the overall requirements and content clauses of the personnel promotion policy related to "urban doctors serving in rural areas" satisfactory from a macro perspective (including but not limited to the requirement for urban doctors to spend a certain number of days in the countryside before promotion)? [Single choice question]

○ Very satisfied

○ Quite satisfied

○ Basic satisfaction

○ Not quite satisfied

○ Very dissatisfied

64. Do you think there is still room for modification in the personnel promotion policy related to "city doctors serving in rural areas" to adapt to the situation in your unit? [Single choice question]

○ There is ample room for significant modifications and improvements.

○ There is some room for modification and improvement.

○ There is a small amount of room for modifications and improvements.

○ The policy is good, no need to modify.

65. How do you think the policy of "city doctors serving in rural areas" should be modified more securely? [Multiple choice]*

□ Remove promotion requirements and allow more doctors to volunteer to go to rural areas.

□ Number of days in the countryside revised.

□ The policy requires mandatory relaxation of doctors' prescription rights.

□ Ensuring smooth communication channels in the HR department makes it easier for doctors whose specialties do not match.

□ Expand the choices for city doctors, not limited to just going to the countryside.

□ Enhance the two-way learning channels and encourage rural doctors to study in cities.

□ Expand the scope and content of services

□ Others _________________*

Dependent on the 1st; 2nd; 3rd options of question 64.

1. Do you think that urban doctors serving in rural areas helps to improve the medical capabilities of the local healthcare institutions (comparable to Beijing's rural medical institutions)? [Multiple choice]

○ Great improvement.

○ a significant improvement

○ General

○ There is a slight improvement.

○ No improvement.
